# University of Maryland Dental Department

**Published:** 1890-02

**Authors:** 


### Editorial, Etc.

UNIVERSITY OF MARYLAND DENTAL DEPARTMENT. —The annual commencement of this institution was held March 19th, 1890., at the Lyceum Theatre, Baltimore, at 8 P. M., in the presence of a large and brilliant assemblage of ladies and gentlemen.

The Music was rendered by Prof. W. F. Thiedes' orchestra.

The Mandamus from the Board of Regents was read by Prof. Ferdinand J. S. Gorgas, the Dean, who announced the following graduates, upon whom the degree of Doctor of Dental Surgery was conferred.

| | |
|---|---|
| David Aiken - - - - | South Carolina |
| J. Maurice Ayer - - - - | North Carolina |
| John R. Berry - - - - - | Virginia |
| William E. Beachley - - - - | Maryland |
| William C. Boswell, Jr. - - - | Maryland |
| Charles T. Breedlove - - - - | Arkansas |
| William Stevens Brown, Jr. - | South Carolina |
| Oscar Frank Byrd - - - - | Virginia |
| Stuart Cassard - - - - - | Maryland |
| William H. Collins - - - - | Virginia |
| St. George T. Craig - - - - | Kentucky |
| Wray Wythe Davis - - - - | Virginia |
| L. Thornwell Emerson - - - | Brazil, S.A |
| Charles Felker - - - - | New York |
| Henty J. Fenn - - - - - | New York |
| Isaac Watts Furman - - - - | New York |
| Harvey Edward Glatfelter - - | Pennsylvania |
| Charles Carroll Graham - - - | Texas |
| J William Graves - - - - - | Missouri |
| N. Robert Hubbard - - - | Pennsylvania |
| Aaron Victor Huntzbery - - - | Maryland |
| W. Spry Hurlock - - - - | Maryland |
| Robert L. Harley - - - | South Carolina |
| William A. King - - - - - | Canada |
| Daniel O. M. LeCron - - - - | Iowa |
| John L. Luke - - - - - - | Virginia |
| J. Henry Marchant - - - - | Virginia |
| Otis H. McDonald - - - - - | Georgia |
| James Elijah McNeal - - - - | Maryland |

| | |
|---|---|
| Thomas P. Meyer - - - | Pennsylvania |
| Charles Mezger, Ph. G - - - | Maryland |
| Laurent S. Mitchell - - - - | New Jersey |
| William W. Morgan - - - | New Jersey |
| Charles Grant Myers - - - - | New York |
| George Harkness Perrin - - - - | Canada |
| Williams Adams Pressley - - | North Carolina |
| Charles B. Renoe - - - - - | Missouri |
| Halsey G. Steer - - - - | New York |
| Sylvanus Claud Sykes - - - - | Maryland |
| F. Foster Todd - - - - | Maryland |
| Charles B. Whelpley - - - - | Canada |
| Charles Edmund Wogan - - | Pennsylvania |
| George Woolsey - - - - | California |
| Clarence Angelo Wright - - | New Hampshire |

The address to the graduates was delivered by Rev. Dr. Elbert S. Todd, of Grace M. E. Church, and the class oration by Harvey E. Glatfelter, D. D. S. The number of matriculates for the session of 1889–90, was *one hundred and thirty-five.*

The award of the following prizes was also announced by the Dean, Prof. Gorgas:

University prize, for the highest number of votes at final examinations, Harvey E. Glatfelter, of Pennsylvania; honorable mention, George H. Perrin, of Canada.

S. S. White, 1st prize, dental engine, for the best lower set of gum teeth on metal, Daniel O. M. Le Cron, of Iowa; honorable mention, Charles Felker, of New York.

Snowden & Cowman, prize, set of Chapin A. Harris' forceps, for the best two fillings of cohesive and non-cohesive gold, George Woolsey, of California; honorable mention, Wray Wythe Davis, of Virginia.

Francis Arnold, prize, set of forceps, for best full upper set of continuous gum, Harvey E. Glatfelter, of Pennsylvania; honorable mention, Robert L. Harley, of South Carolina.

James Hart, prize, gold medal, for the best partial set on metal, John H. Marchant, of Virginia; honorable mention, St. George T. Craig, of Kentucky.

S. S. White, 2d prize, set of Varney's pluggers, for the best set on celluloid base, Charles Felker, of New York; honorable mention, James E. McNeal, of Maryland.

Prof. James H. Harris, prize, gold medal, for the best filling of non-cohesive gold, Harvey E. Glatfelter, of Pennsylvania; 1st honorable mention, J. Wm. Graves, of Missouri; 2d honorable mention, J. Maurice Ayer, of North Carolina.

Prof. F. J. S. Gorgas, prize, gold medal, for the best contour filling of cohesive gold, Oscar F. Byrd, of Virginia; honorable mention, Aaron V. Huntzbery, of Maryland.

Dr. Jno. C. Uhler, prize, gold medal, for the best set on vulcanite base, William E. Beachley, of Maryland; honorable mention, Charles Felker, of New York.

Dr. Isaac H. Davis, prize, gold medal, for the best crown-and-bridge-work, Henry J. Fenn, of New York, 1st honorable mention, Daniel O. M. Le Cron, of Iowa; 2d honorable mention, George Woolsey, of California.

The Fish Class prize, Snowden & Cowman vulcanizer, for the best specimen set of teeth, Charles Felker, of New York.

Special Class prize, gold headed cane, for the best gold filling in specimen tooth, Harvey E. Glatfelter, of Pennsylvania; 1st honorable mention, Oscar F. Byrd, of Virginia; 2d honorable mention, Charles Felker, of New York.

